# Functionalized Controlled Porous Glasses for Producing Radical-Free Hyperpolarized Liquids by Overhauser DNP

**DOI:** 10.3390/molecules27196402

**Published:** 2022-09-28

**Authors:** Raphael Kircher, Sarah Mross, Hans Hasse, Kerstin Münnemann

**Affiliations:** Laboratory of Engineering Thermodynamics, University of Kaiserslautern, 67663 Kaiserslautern, Germany

**Keywords:** Overhauser DNP, radical immobilization, benchtop NMR, continuous-flow NMR

## Abstract

Overhauser dynamic nuclear polarization (ODNP) can be used as a tool for NMR signal enhancement and happens on very short time scales. Therefore, ODNP is well suited for the measurement of fast-flowing samples, even in compact magnets, which is beneficial for the real-time monitoring of chemical reactions or processes. ODNP requires the presence of unpaired electrons in the sample, which is usually accomplished by the addition of stable radicals. However, radicals affect the nuclear relaxation times and can hamper the NMR detection. This is circumvented by immobilizing radicals in a packed bed allowing for the measurement of radical-free samples when using ex situ DNP techniques (DNP build-up and NMR detection happen at different places) and flow-induced separation of the hyperpolarized liquid from the radicals. Therefore, the synthesis of robust and chemically inert immobilized radical matrices is mandatory. In the present work, this is accomplished by immobilizing the radical glycidyloxy-tetramethylpiperidinyloxyl with a polyethyleneimine (PEI) linker on the surface of controlled porous glasses (CPG). Both the porosity of the CPGs and also the size of the PEI-linker were varied, resulting in a set of distinct radical matrices for continuous-flow ODNP. The study shows that CPGs with PEI-linkers provide robust, inert and efficient ODNP matrices.

## 1. Introduction

NMR spectroscopy is a very versatile analytical method which is used in many areas of science, but also in clinical medicine and industry. Ever since its discovery, efforts have been made to increase the intrinsically low sensitivity of NMR experiments. This includes lively research on hyperpolarization methods for NMR signal amplification, such as para-hydrogen induced polarization (PHIP) [1,2,3], optical pumping [4,5], and dynamic nuclear polarization (DNP) [6,7,8]. DNP relies on transferring the intrinsic higher thermal equilibrium polarization of electron spins to surrounding nuclear spins. Thus, a prerequisite for DNP is the existence of unpaired electrons in the sample, which is mostly accomplished by the addition of stable organic radicals. In liquids, polarization transfer is governed by the Overhauser effect. This is a relaxation-mediated process, which is most efficient when the longitudinal relaxation time $T_{1}$ of the nuclear spin is dominated by interactions with electron spins. ODNP happens on very short time scales, which makes this technique ideally suited for the continuous hyperpolarization of flowing liquids [9,10,11,12,13,14,15,16,17,18,19,20].

Currently, Overhauser DNP (ODNP) is increasingly used in combination with compact NMR instruments [20,21,22,23,24] because ODNP units are rather small and add only moderately to the weight and size of the overall NMR system. Compact NMR systems are mainly based on permanent magnets and allow a very flexible use of NMR spectroscopy because a dedicated laboratory infrastructure and cryogenic coolants are not needed. Many applications can profit from compact NMR spectrometers, which provide fast on-spot sample characterization, from standard laboratory analysis to online process monitoring. The use of permanent magnets, however, limits the maximal achievable magnetic field strength and, therefore, the signal-to-noise ratio (SNR) that, in turn, restricts applications. The situation becomes even worse when flowing samples have to be investigated, as it is often the case in reaction and process-monitoring applications [25,26,27,28,29,30,31,32,33]. The compact design of benchtop NMR spectrometers leads to short residence times of the flowing sample in the magnetic field of the spectrometer, resulting in inefficient polarization build-up and, thus, poor signal intensity.

The typical ODNP experiment under continuous flow is carried out as follows: The flowing sample passes first the ODNP unit containing a packed bed of immobilized radicals for hyperpolarization build-up before it enters the NMR spectrometer. The immobilization of radicals is necessary in these setups because the presence of free radicals reduces the nuclear $T_{1}$ of the liquid and thereby also the lifetime of the accomplished hyperpolarization during the transport time from the ODNP unit to the NMR detector. Consequently, a major part of the enhanced signal would be lost due to rapid $T_{1}$ relaxation during the transport time if dissolved radicals were used. Moreover, radicals affect also the transverse relaxation time $T_{2}$, resulting in line broadening of the NMR signals if present during NMR detection. Dorn et al. [9,10,34] were the first to present and optimize ODNP on continuously flowing samples. They introduced the abbreviation SLIT-DNP (solid/liquid intermolecular transfer) because they used radicals immobilized on silica-based solids as ODNP polarizations agents. Silica radical matrices [9,10,11,12] were studied by using various linker structures for coupling TEMPO radicals on the surface of the solids. Linkers that provide a large distance between the radicals and the solid surface worked best in these studies. More recently, other groups have employed continuous-flow ODNP for clinical purposes, i.e., for the generation of a continuous stream of hyperpolarized water as a MRI contrast agent, by using hydrogel-based polarization matrices [13,16,35,36,37]. In these approaches, mainly sepharose was used as a hydrogel matrix for TEMPO radicals. The Han group further optimized sepharose-TEMPO matrices by introducing an additional polyelectrolyte linker, which led to a strong increase in the ODNP signal enhancement [13,14].

Although the use of immobilized radical matrices is essential for continuous-flow ODNP, only a very small number of radical matrices have been described in the literature so far. The systems can roughly be divided into two groups, depending on the used matrix: (1) based on silica and (2) based on hydrogels, with sepharose as the most prominent example. For applying continuous-flow ODNP for process monitoring studies, radical matrices should fulfill the following requirements: sufficient radical loading, high mobility of radicals, easy access of the target molecules to the radicals, chemical stability, compatibility with a wide range of solvents, temperature stability, pH stability, long-term stability, and a morphology that enables using them in packed beds with favorable flow characteristics. Thus, hydrogels, such as sepharose, have several drawbacks, e.g., poor chemical stability, strong temperature, pH, and pressure dependence, and limited applicability in non-aqueous solutions. In water-free systems, the radical–target molecule contact is substantially diminished due to poor swelling of the hydrogel, resulting in bad ODNP efficiency. Silica-based radical matrices, on the other hand, fulfill most of the above-mentioned requirements. However, if porous solids, such as silicas, are used for radical immobilization, special care has to be taken that materials are chosen for which the influence of the pore walls on the $T_{1}$ relaxation time of the liquid is small. McCarney et al. [16] showed that the ODNP efficiency of commercially available spin labeled silica matrices (with extremely high radical loadings of up to 900 mM) is poor because extra modes of proton relaxation in porous materials already decrease the native $T_{1}$ time by around 90%, even without immobilized radicals. These matrices had very small pore sizes of 6 nm and 10 nm, thus $T_{1}$ relaxation via the pore walls was extremely efficient and the electron-driven part of the nuclear $T_{1}$ was not dominant. To avoid this effect, porous matrices with larger pore sizes have to be chosen for ODNP applications. Unfortunately, this reduces the specific surface area of the porous materials and, thus, the number of possible binding sites for immobilization so that it is hard to achieve sufficiently high radical loadings, which are required for a high ODNP efficiency.

Therefore, we have synthesized new silica-based radical matrices with polyethylene-imine (PEI) linkers and immobilized the nitroxide radical 4-glycidyloxy-2,2,6,6-tetramethyl- piperidine-1-oxyl (GT) for continuous-flow ODNP applications and tested their performance. The aim was to create materials suitable for process monitoring applications. We used controlled pore glasses (CPGs) as the matrix, with large pore sizes of 50, 100, and 200 nm, because they fulfill the above-mentioned design criteria: they are chemically inert, stable in a wide temperature and pH range, have a narrow pore size distribution, provide fairly weak wall relaxation and have good flow characteristics. However, the number of binding sites on these CPGs for the attachment of radicals is not sufficient for obtaining a good ODNP. Therefore, we developed a synthesis strategy in which we first attach PEI to the binding sites of the CPGs. The polymer PEI in turn provides many binding sites for the covalent attachment of radicals. PEI is also known for the high-performance immobilization of bio-catalysts [38,39,40,41]. In the last step, the nitroxide radical GT was immobilized on the PEI-grafted CPGs. Both the porosity of the CPGs and the size of the PEI-linker were varied in subsequent syntheses. Furthermore, the radical–target molecule contact can be varied in these systems by adjusting the pH, because in the protonated form, at low pH, the PEI-linker is stretched. The stretched form leads to improved spatial expansion of the PEI-linker and, therefore, to an easier accessibility of the immobilized radicals. This approach combines the ideas of Dorn et al. [12] (large radical–surface distance) and Han et al. [13] (using a polyelectrolyte linker) for optimal ODNP efficiency and adds on top of this the possibility of easily achieving high radical loadings in the systems.

This paper is organized as follows: First we give a short introduction in the basic Overhauser DNP theory, as this is mandatory to understand the influence of the different factors impacting ODNP efficiency. Thereafter, the synthesis of CPG radical matrices is described, and the ODNP setup used for the studies is explained. In the Results and Discussion section, the results of the experimental studies on the influences of different factors that determine the ODNP efficiency are presented and compared to the literature values, where possible. This comprises the influence of the radical loading, which was studied by EPR spectroscopy, and the ODNP leakage factor, which was determined by $T_{1}$ inversion recovery measurements. ODNP experiments in static contact with the radical matrix and in continuous flow in which the microwave power was varied are presented as well as the results from experiments in which the pH was varied to study the influence of the conformation of the PEI-linker. Finally, we conclude and provide an outlook on possible applications.

## 2. Overhauser Dynamic Nuclear Polarization

ODNP relies on the transfer of polarization of electron spins to surrounding nuclear spins and was already assumed by Albert Overhauser in 1952 [42,43,44]. The polarization transfer is driven by microwave irradiation of electrons and electron–nuclear hyperfine coupling, which is of a dipolar nature for protons and nitroxide radicals. The detailed theory of ODNP is given elsewhere [45,46], and only essential relations are introduced below. The ODNP enhancement (*E*) of the NMR signal is the ratio of signals of hyperpolarized measurements $I_{Z}$ (microwave on) and those of thermally polarized measurements $I_{0}$ (microwave off) and is calculated as given by Equation (1):

(1) $$E\;=\;\frac{I_{Z}}{I_{0}}\;=\;1-\xi sf\frac{|\gamma_{S}|}{\gamma_{I}},$$

where *s* is the saturation factor, ξ is the coupling factor, and *f* is the leakage factor. The constant $\gamma_{I}$ denotes the gyromagnetic ratio of the nucleus and $\gamma_{S}$ that of the electron. The maximal theoretical enhancement for ${}^{1}$H ODNP is about 660 (i.e., the ratio of $\gamma_{I}$ and $\gamma_{S}$), but the other parameters are the limiting factors. The coupling factor ξ, which is a measure of the hyperfine interaction between the electron and nuclear spin, ranges between −1.0 for pure scalar coupling and 0.5 for pure dipolar coupling. For the nitroxide-based radical matrix that was used in the present work with ${}^{1}$H ODNP, the dipolar interaction is dominant [45], thus the maximum achievable enhancement is limited to about −330 [47]. The coupling factor ξ depends on the temperature and magnetic field strength [45,46,48] and can be determined with several methods: (1) molecular dynamic simulations can be performed, which, however, require modeling assumptions and are computationally expensive [49,50,51], especially in SLIT-DNP; (2) $T_{1}$ relaxation dispersion data can be used [52,53,54,55]; and (3) recalculation from measured ODNP enhancements can be done, if the other ODNP parameters are known. The latter approach provides only a rough estimation because of temperature effects occurring during microwave irradiation. The saturation factor *s* describes the efficiency of the saturation of the electron spins and strongly depends on the applied microwave power. It ranges between 0 and 1. It can be determined with several methods: (1) pulsed ELDOR measurements can be performed for precise determination [56,57,58]; (2) Prisner et al. used the influence of the paramagnetic shift by applied microwave power to calculate the saturation factor [53,59,60]; (3) and recalculation can be performe dwith the ODNP data, if other ODNP parameters are known. The leakage factor *f* denotes the effectiveness of electron-driven nuclear spin relaxation and ranges also between 0 and 1. This ODNP parameter is directly accessible via NMR and is calculated from

(2) $$f\;=\;1-\frac{T_{1}^{\mathrm{RM}}}{T_{1}},$$

where $T_{1}^{\mathrm{RM}}$ denotes the longitudinal relaxation time of the nuclear spin in static contact with the radical matrix and $T_{1}$ the longitudinal relaxation time in static contact with the PEI-grafted CPG matrix, but without immobilized radicals. Reaching a value of *f* = 1 indicates the desired dominant relaxation via the electron spins. This is achieved at sufficiently high radical concentrations, e.g. for the widely used 4-hydroxy-TEMPO radical for concentrations of around 20 to 30 mM.

## 3. Materials and Methods

### 3.1. Radical Matrix Design

**Figure 1:**
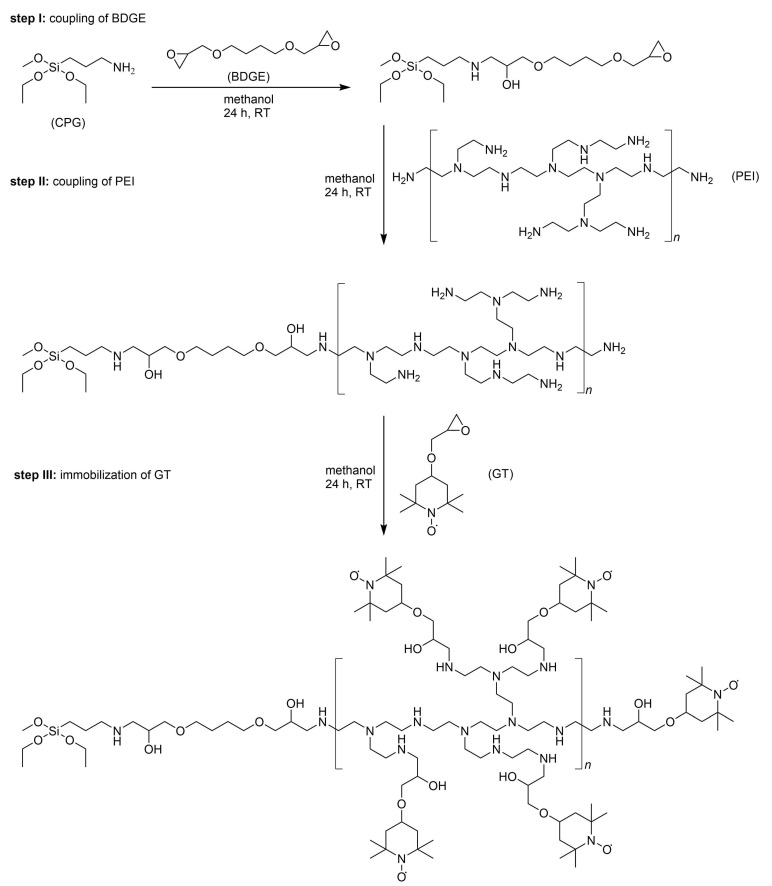
Scheme of the three-step immobilization procedure: (1) coupling of BDGE on aminopropyl-functionalized CPGs; (2) coupling of PEI on glycidyl-functionalized CPGs; (3) immobilization of GT. In this work, radical matrices with immobilization of GT directly on the surface of aminopropyl-functionalized CPGs (only step III) and with immobilization indirectly on CPGs with BDGE-linker and PEI-linker were synthesized (step I to step III). All reactions were solely performed at room temperature (RT) in methanol.

The radical matrices designed in the present work consist of the nitroxide radical glycidyloxy-tetramethylpiperidinyloxyl (GT) immobilized on aminopropyl-functionalized CPGs and were synthesized in our laboratory. In addition, to immobilize GT directly on aminopropyl-functionalized CPGs, the PEI-linker and the intermediate linker 1,4-butanediol diglycidyl (BDGE) ether were used as coupling agents. Figure 1 shows a simplified immobilization scheme that is divided into three steps: step I—coupling of BDGE-linker on aminopropyl-functionalized CPGs; step II—coupling of PEI on BDGE-grafted CPGs; and step III—immobilization of GT. In the first case of immobilizing GT directly on the CPG surface, the first two steps in Figure 1 were omitted, and the nitroxide radical was immobilized in step III on aminopropyl-functionalized CPGs. For the second case of immobilizing GT indirectly with PEI-linker and BDGE-linker, the intermediate linker BDGE was covalently bound to provide glycidyl-functionalized CPGs in step I, and afterwards PEI was added in step II. All reactions were solely carried out at room temperature (RT) in methanol.

Aminopropyl-functionalized CPGs with three different pore sizes (50, 100, and 200 nm) were used as starting materials with surface areas according to the manufacturer’s data of 25 m${}^{2}$ g${}^{-1}$, 51 m${}^{2}$ g${}^{-1}$, and 69 m${}^{2}$ g${}^{-1}$. PEI-linker with different molecular masses, 800 g mol${}^{-1}$ (PEI800) and 25,000 g mol${}^{-1}$ (PEI25000), were attached to the CPGs. The coupling reactions in the presented immobilization procedure of GT take place exclusively with the reaction of the epoxy- and amino groups in all steps. A distinction in the matrix design is achieved with the following ways of radical immobilization: (i) directly on the surface of the CPGs; (ii) indirectly with short PEI-linker (PEI800); and (iii) indirectly with long PEI-linker (PEI25000). In this way, nine different radical matrices were synthesized. The detailed synthesis procedure is described in the Appendix A, and the procedure to quantify the amount of immobilized GT by EPR spectroscopy is provided in the Supporting Information.

### 3.2. ODNP Set-Up

**Figure 2:**
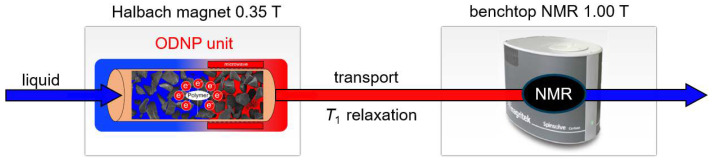
Scheme of the set-up for continuous-flow benchtop NMR measurements with ODNP unit. ODNP is performed at 0.35 T in a Halbach magnet. ODNP measurements are performed in two ways: (1) static ODNP with hyperpolarization directly in the Halbach magnet that is equipped with an ENDOR probehead for simultaneous microwave irradiation and NMR detection, and (2) continuous-flow ODNP, where hyperpolarization is accomplished in the Halbach magnet and NMR detection in the 1.00 T benchtop NMR spectrometer (transport distance is around 0.5 m). A HPLC pump is used to set up the liquid flow.

The ODNP-enhanced continuous-flow NMR set-up is shown in a simplified overview in Figure 2. The liquid passes first a packed bed with the radical matrix that is placed in a 0.35 T Halbach magnet [20], which is equipped with an ENDOR probehead. Here, microwave irradiation and, thus, ODNP polarization build-up is accomplished. The liquid flows then through a PEEK capillary (250 μm inner diameter) to a 1 T benchtop NMR spectrometer, in which the capillary simply passes the bore and is used as flow probe.

Besides continuous-flow experiments [20], also static experiments were carried out [61]. Static ODNP measurements were performed using the ENDOR probehead in the Halbach magnet also as NMR detector, i.e., without using the benchtop NMR spectrometer. Experimental procedures are described in the Appendix B.

## 4. Results and Discussion

### 4.1. Radical Matrix Characterization

Table 1 lists all synthesized radical matrices in this work, CPG-GT (without PEI), CPG-PEI800-GT, and CPG-PEI25000-GT that were used in ODNP-enhanced NMR spectroscopy. Radical loadings of all radical matrices are provided which were measured by EPR spectroscopy and are listed in Table 1.

The radical loadings were calculated by comparison of the EPR integrals with aqueous solutions of GT of known concentrations (see Supporting Information, where also representative EPR spectra are provided). The radical loading for each set of CPGs increases with decreasing pore size. This trend is expected as also the surface area and, thus, the binding capacity of the CPG based materials increases with decreasing pore size. Furthermore, a significant increase of the immobilized radical loading is achieved by using PEI. The desired radical loading (of about 30 mM) for ODNP was achieved with radical matrices of the type CPG-PEI25000-GT with a pore size of 100 and 50 nm.

Table 2 lists $T_{1}$ values of acetonitrile and water in static contact with the radical matrices and with the plane matrix without immobilized radicals, CPG, CPG-PEI800, and CPG-PEI25000 that were used to study the relaxation behavior dependent on the pore size of CPGs and the size of the PEI-linker.

The next step is to investigate the efficiency of CPGs for decreasing $T_{1}$ and to finally calculate ODNP leakage factors. Therefore, standard inversion recovery measurements were carried out in a 1 T benchtop NMR spectrometer with pure water and pure acetonitrile in two ways: (1) in static contact with the radical matrix ($T_{1}^{\mathrm{RM}}$); and (2) in static contact with the plane matrix, without immobilized radicals ($T_{1}$). Acetonitrile and water were chosen as target molecules because of their different $T_{1}$ values. As stated before, it is mandatory for high ODNP efficiency that the $T_{1}$ of nuclear spins are dominated by the interaction with electron spins, i.e., $T_{1}^{\mathrm{RM}}$ have to be much smaller than $T_{1}$. This condition is best fulfilled if the influence of wall relaxation and relaxation via the polymer PEI is small for target molecules. Table 2 shows that the $T_{1}$ values of bulk water (native $T_{1}$ = 3.0 s) is decreased by 42% by the plane CPG matrix with the smallest pore diameter of 50 nm and by around 31% for the plane CPG matrix with the largest pore diameter of 200 nm. Thus, also in the present study, an increasing effect of wall relaxation with decreasing pore size was found, as it becomes also obvious from the data of bulk acetonitrile (native $T_{1}$ = 4.0 s).

Moreover, Table 2 shows that also the coupling of BDGE and PEI to the surface of the CPGs results in a slight decrease in the $T_{1}$ values of water and acetonitrile. This effect becomes stronger with the increasing size of the PEI-linker, but is still moderate, compared to the influence of the pore walls. Thus, the $T_{1}$ reduction in our systems is much less pronounced than in the silica-based systems with very small pore sizes described in the literature [16] (pore size of 6 and 10 nm, $T_{1}$ drop by wall relaxation of 90%). $T_{1}$ in our systems remain long enough to allow for sufficient electron-driven relaxation. It is clearly shown that with increasing radical loading, a very substantial decrease in the relaxation times of water and acetonitrile is achieved, cf. Table 1 and Table 2, as it is necessary for high ODNP efficiency; the best results were achieved with the radical matrix CPG-PEI25000-GT with the smallest pore size of 50 nm and the highest amount of immobilized GT.

The positive trend in the $T_{1}$ data manifests itself in the calculation of the leakage factors, cf. Equation (2). Leakage factors are close to 1 if nuclear $T_{1}$ is dominated by unpaired electrons, which is achieved at sufficiently high radical loadings (20–30 mM; we demonstrate this in the Supporting Information for various dissolved TEMPO radicals in water and acetonitrile). For our CPGs, we achieved these high radical loadings and optimal leakage factors only for CPG-PEI25000-GT with radical loadings ranging from 12 to 37 mM. For this reason, we decided to work only with the CPG-PEI25000-GT materials in the subsequent experiments. The leakage factors for these systems are listed in Table 3 and show promising values of up to 0.94 for both solvents. This means that our strategy of choosing fairly large pore sizes of the CPGs and providing additional radical binding sites via introduction of the PEI-linkers worked well. The leakage factors of all other synthesized materials in acetonitrile and water are shown in the Supporting Information.

### 4.2. Static ODNP

**Figure 3:**
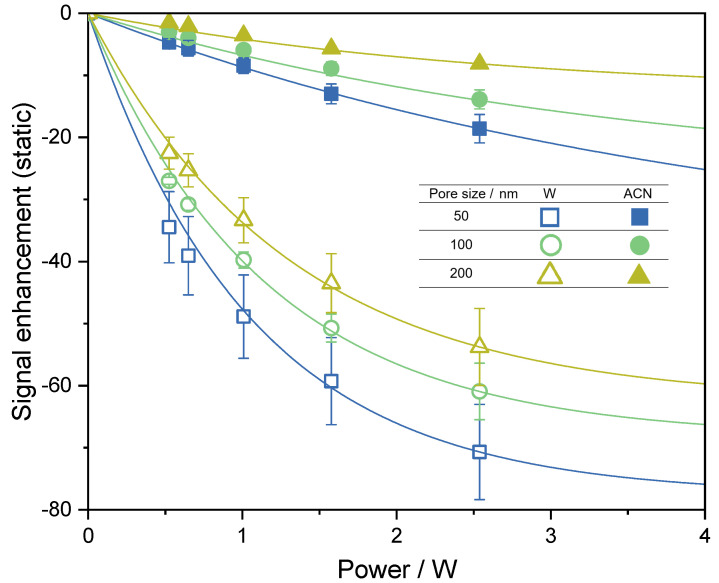
Results from static ODNP measurements with water (W) and acetonitrile (ACN) obtained with CPG-PEI25000-GT of different pore sizes and different applied microwave power values. The symbols are experimental results and were obtained as the arithmetic mean of the measurements of five individual samples. The error bars indicate the standard deviation. Solid lines: extrapolation to infinite microwave power [45,62].

The performance of the synthesized radical matrices in ODNP was studied with static ODNP-enhanced NMR measurements with varying microwave power using radical matrices of the type CPG-PEI25000-GT directly in the ENDOR probehead in microcapillaries. The results are shown in Figure 3. Signal enhancement was calculated with the integral of the hyperpolarized signal (microwave on) relative to the integral of the conventional thermally polarized NMR signal (microwave off). Only low microwave power levels in the range of around 0.5 to 2.5 W were used because otherwise, significant sample heating would be introduced, which results in increasing coupling factors [48], and often leads to misinterpreted results.

Figure 3 shows that high absolute ODNP enhancements were achieved with studied CPGs, especially with those with the lowest pore size (50 nm), for which the highest measured absolute values were about −20 for acetonitrile (ACN) and −70 for water (W). The large differences in the enhancement values measured for water and acetonitrile are explained by the different hyperfine interactions of the methyl group of acetonitrile and the water protons with the TEMPO radical, which is in line with the findings for $T_{1}^{\mathrm{RM}}$, cf. Table 2. TEMPO radicals are particularly well suited for the hyperpolarization of water. From ODNP data with increasing microwave power, $E_{\max}^{\mathrm{static}}$ values for the enhancement at infinite microwave power were extracted by exponential extrapolation [45,62]. These $E_{\max}^{\mathrm{static}}$ values in combination with the measured leakage factor values can be used to calculate an estimate of the ODNP coupling factor ξ by assuming that the saturation factor *s* is 1 [13,63]. The corresponding values are listed in Table 3. These values for the coupling factors ξ are conservative estimations, as the saturation factor *s* is usually lower than 1. The coupling factor for water obtained in this way is about 0.12, whereas that for acetonitrile is only about 0.05; this shows again that TEMPO radicals are particularly well suited for ODNP in water. Furthermore, a temperature effect leads to a higher coupling factor of water compared to acetonitrile: the dielectric properties of water and acetonitrile are fundamentally different, which leads to a substantially stronger heat uptake under microwave irradiation for water than for acetonitrile, which, in turn, leads to a higher coupling factor for water. Therefore, the difference in the isothermal coupling factors between water and acetonitrile is likely to be smaller than suggested by the values in Table 3. In the literature, some values for static ODNP measurements of water with immobilized TEMPO systems are available for comparison, as many groups focus on aqueous systems. The Han group worked with two different sepharose-based matrices for hyperpolarization of water [16]. The first matrix was synthesized by the coupling of TEMPO radicals directly on sepharose and provided a coupling factor of 0.07 and an $E_{\max}^{\mathrm{static}}$ value of −42. For the second matrix, an additional polyvinylimidazole polyelectrolyte linker was used; an improved coupling factor of 0.22 was achieved and an $E_{\max}^{\mathrm{static}}$ value of −122 was obtained, which is above the $E_{\max}^{\mathrm{static}}$ value obtained in the present work. The main difference is that the polyvinylimidazole linker is a polyelectrolyte, which exhibits not only a favorable interaction with water, but is also present in a stretched conformation in water at pH 7, leading to improved radical–target molecule contact. However, the PEI-linker used in our systems can be easily converted in a polyelectrolyte by simply lowering the pH. PEI is essentially uncharged at about pH 7; however, upon lowering the pH, the amine groups are protonated and PEI obtains a positive net charge. This is expected to lead to stretched conformation [64], and hence, to an improved accessibility of the radicals.

**Figure 4:**
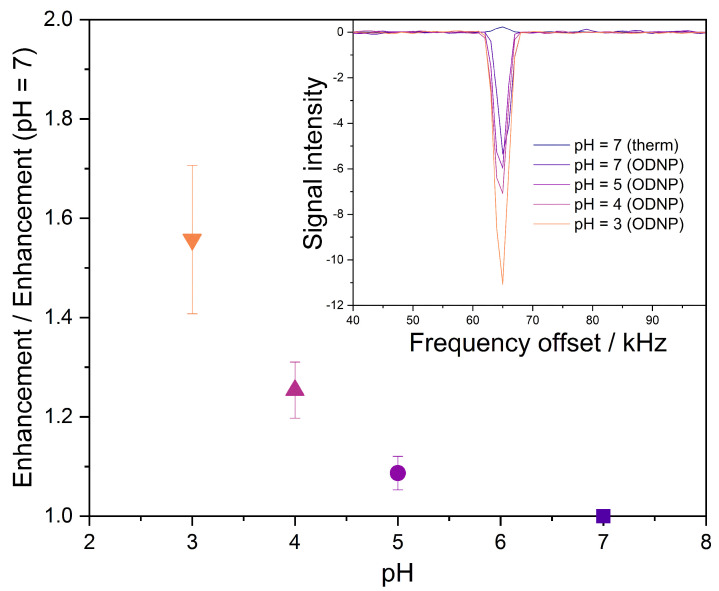
Results from static ODNP-enhanced ${}^{1}$H NMR measurements of water with CPG-PEI25000-GT (pore size 200 nm) at different pH. The symbols are experimental results and were obtained as the arithmetic mean of five individual samples. The error bars indicate the standard deviation. Insert: thermally polarized (therm, acquired with 16 scans) and hyperpolarized (ODNP, single-scan) ${}^{1}$H NMR spectra.

To test this hypothesis, we performed static ODNP-enhanced NMR measurements of water at different pHs using CPG-PEI25000-GT with a pore size of 200 nm. The radical matrix with the highest pore size was chosen for these experiments in order to observe the stretching effect of the PEI-linker at lower pHs undisturbed from size restrictions introduced by the pore walls. The 0.2 M solution of hydrochloric acid was used for adjusting the pH in the solutions to 3, 4, 5, and 7. The results of the corresponding measurements are shown in Figure 4.

The hypothesis is fully confirmed by the results shown in Figure 4. Going from pH 7 to pH 3 the absolute enhancement increases by more than 50%. The enhancements measured for pH 3 was about −100 and, hence, in the range of the values achieved by with sepharose-based materials that are grafted with polyvinylimmidazole [13]. Furthermore, the PEI-linker that was used in the present work was branched, which limits the positive effect of stretching on the accessibility. However, our results clearly show that CPG radical matrices tolerate acidic conditions, which is not the case for sepharose-based matrices. This chemical resistance is highly beneficial for process-monitoring applications.

### 4.3. Flow ODNP

**Figure 5:**
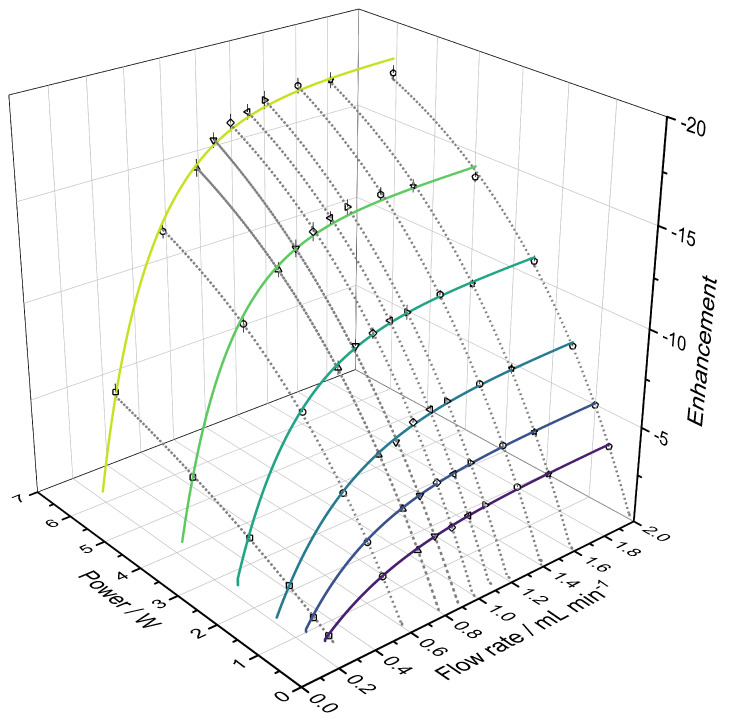
Results from continuous-flow ODNP measurements with water obtained with CPG-PEI25000-GT (pore size 50 nm) versus applied microwave power and flow rate. The symbols are experimental results and were obtained as the arithmetic mean of the results of three individual samples. Solid lines: guide to the eye. Dashed lines: exponential fit [45,62].

We have tested the ODNP performance of the new CPGs in continuous-flow ODNP experiments with water and acetonitrile. Continuous-flow ODNP experiments have the advantage that sample heating effects are significantly reduced compared to static experiments because the irradiated sample volume is continuously exchanged. However, the microwave operation times are much longer in continuous-flow experiments than in the static experiments described before, which can result in significant heating of the microwave resonator itself and a corresponding frequency drift. Thus, the microwave resonator was actively cooled with cold nitrogen gas in our continuous-flow ODNP experiments. The temperature of the ENDOR probehead was measured by a platinum resistance thermometer and kept at about 288 K by adjusting the temperature of the nitrogen feed, which was controlled by electric heating [20]. Figure 5 shows results from continuous ODNP measurements with CPG-PEI25000-GT (pore size 50 nm) with water, in which both the microwave power and the flow rate were varied. The highest flow rate corresponds to a superficial flow velocity of about 0.7 m s${}^{-1}$. The corresponding results for acetonitrile are shown in the Supporting Information and confirm the trends from Figure 5.

The signal enhancement in continuous-flow ODNP experiments was calculated from the integral of the hyperpolarized signal measured in the 1 T benchtop NMR (microwave on) at a certain flow rate relative to the integral of the thermally polarized benchtop NMR signal at a flow rate of 0.25 mL min${}^{-1}$ (microwave off). At this flow rate, the first hyperpolarized signals were detected in continuous-flow ODNP measurements independent of applied microwave power. Thermal polarization of water in the benchtop NMR spectrometer is about three times larger than the thermal polarization in the 0.35 T Halbach magnet. Therefore, the absolute ODNP enhancement is expected to be three times lower than in the powersweep data shown in Figure 3. The enhancement values observed in flow experiments are slightly smaller than this, which is caused by polarization losses due to $T_{1}$ relaxation during the transport of the hyperpolarized water from the ODNP unit to the NMR detection site, cf. Figure 2. $E_{\max}^{\mathrm{static}}$ values of water were reached at a microwave power of about 6 W (cf. Figure 3 and Table 3) in the static powersweep experiments, whereas the extrapolation of the data shown in Figure 5 indicates that in continuous-flow experiments about 15 W are needed. The differences to the static powersweeps are attributed to the different saturation efficiency of the sample. Static experiments were performed in a glass microcapillary and continuous-flow experiments in a dedicated container made of PEEK to store the CPG radical matrix. In addition, coupling factors in continuous-flow ODNP are less amplified by increasing temperature during microwave irradiation.

Figure 5 shows that a large sensitivity improvement in continuous-flow benchtop NMR spectroscopy was accomplished by ODNP. This is especially important in the fast flow regime, where the thermal NMR signal almost completely vanishes due to very short prepolarization times. The overall volume in our set-up is less than 2 mL. NMR measurements were performed directly on the peek capillary, resulting in a small sample volume of about 0.5 μL in the NMR coil, but still NMR spectra with very good SNR were recorded in single-scan acquisition at fast flow rates. Finally, the long-term stability of the synthesized CPG radical matrices for continuous-flow ODNP was investigated. The radical matrices were stored in different liquids, i.e., acetone, water, diethyl ether, acetonitrile and 3-pentanone, for a period of 50 days, during which the liquid supernatant was analyzed for possible radical leakage by EPR spectroscopy. No radicals were detected in any of the solvents, indicating a good long-term stability of the CPG radical matrices.

## 5. Conclusions

In this work, new CPG radical matrices for producing hyperpolarized radical-free liquids via ODNP are presented. CPGs are robust and chemically inert solid materials that can be used in process monitoring applications. The radical matrices were designed to fulfill the following requirements: sufficient radical loading for high ODNP efficiency, high mobility of radicals and good accessibility of the radicals for the target molecules, chemical inertness, temperature stability, pH stability, long-term stability, and good flow characteristics. The synthesis of the new CPGs radical matrix is simple and is accomplished in three steps by using non-hazardous chemicals. A set of six different radical matrices was synthesized by using CPGs with different pore diameters and two PEI-linkers with different molecular masses for the immobilization of TEMPO radicals. The use of the polymeric PEI-linker is mandatory to increase the number of binding sites for the radicals, which are too low for plane CPGs. The radical loading of the synthesized matrices was measured by EPR spectroscopy and the relaxation behavior of liquids in static contact with these matrices was studied by NMR spectroscopy, allowing for the determination of the ODNP leakage factor. It was demonstrated that the radical loadings achieved with the high-molecular mass PEI-linker are sufficient, with leakage factors of about 0.9. ODNP measurements dependent on microwave power were performed for acetonitrile and water in static contact with these CPG radical matrices in order to obtain information of the maximal possible ODNP enhancements ($E_{\max}^{\mathrm{static}}$) and the ODNP coupling factor ξ. The obtained $E_{\max}^{\mathrm{static}}$ values as well as the coupling factor ξ for water are similar to values that were obtained for sepharose-based systems known from the literature. Moreover, we were able to substantially improve the obtained enhancements simply by lowering the pH in our measurements with water. This is attributed to the fact that the PEI-linker becomes a stretched polyelectrolyte when it is protonated, resulting in improved radical-target molecule contact, which is highly beneficial for ODNP. Our results clearly show that CPGs tolerate acidic conditions which is useful for process monitoring applications. Results from stability tests that were carried out for 50 days indicate a good long-term stability of the new CPG radical matrices in common solvents. The CPG radical matrices were also tested in continuous-flow ODNP experiments, demonstrating that they provide a large sensitivity improvement in continuous-flow benchtop NMR spectroscopy. The active volume in the NMR coil of the benchtop spectrometer is about 0.5 μL, but still satisfying NMR spectra were recorded in single-scan acquisition at fast flow rates with ODNP. The major extension of the accessible range of flow rates of continuous-flow benchtop NMR spectroscopy that is achieved is therefore especially important for the monitoring of very fast processes, where the residence time and volume in bypass lines have to be as small as possible to obtain a high temporal resolution. With CPG radical matrices, we found a robust material that can be used as a packed bed for ODNP applications, which can be easily modified to allow improved hyperpolarization of polar or nonpolar solvents by using various different polymers and radicals.

## Figures and Tables

**Table 1:** Concentrations *c* of immobilized GT on CPGs with pore size *d* of 50, 100 and 200 nm from EPR measurements. Radical matrices were synthesized without linker (CPG-GT), with PEI800-linker (CPG-PEI800-GT), and with PEI25000-linker (CPG-PEI25000-GT).

Radical Matrix	CPG-GT	CPG-PEI800-GT	CPG-PEI25000-GT
*d*/nm		*c*/mM	
50	1.0	7.5	37.4
100	0.3	3.5	31.9
200	0.2	2.3	12.4

**Table 2:** Longitudinal relaxation times $T_{1}$ of water (W) and acetonitrile (ACN) in static contact with CPGs with pore size *d* of 50, 100 and 200 nm at 1 Tesla and 28.5 ${}^{\circ }C$. $T_{1}^{\mathrm{RM}}$ indicates values obtained in contact with the radical matrix, $T_{1}$ indicates values in contact with PEI-grafted CPG matrices without radicals.

Radical Matrix	CPG-GT		CPG-PEI800-GT		CPG-PEI25000-GT	
*d*	$T_{1}^{\mathrm{RM}}$(W)	$T_{1}^{\mathrm{RM}}$(ACN)	$T_{1}^{\mathrm{RM}}$(W)	$T_{1}^{\mathrm{RM}}$(ACN)	$T_{1}^{\mathrm{RM}}$(W)	$T_{1}^{\mathrm{RM}}$(ACN)
/nm	/s	/s	/s	/s	/s	/s
50	0.95	1.85	0.19	0.54	0.08	0.09
100	1.48	2.47	0.34	0.72	0.09	0.14
200	1.99	3.01	0.48	0.97	0.16	0.25
**Matrix**	**CPG**		**CPG-PEI800**		**CPG-PEI25000**	
*d*	$T_{1}$(W)	$T_{1}$(ACN)	$T_{1}$(W)	$T_{1}$(ACN)	$T_{1}$(W)	$T_{1}$(ACN)
/nm	/s	/s	/s	/s	/s	/s
50	1.73	2.78	1.71	2.55	1.27	1.89
100	1.97	2.86	1.77	2.72	1.49	2.33
200	2.08	3.10	1.90	2.94	1.51	2.51

**Table 3:** ODNP parameters of synthesized CPG radical matrices of the type CPG-PEI25000-GT. $E_{\max}^{\mathrm{static}}$ values are calculated from static ODNP measurements that are dependent on microwave power with extrapolation to infinite microwave power. Coupling factors were estimated with the assumption of saturation factors equal to 1 and are also listed.

Pore Size/nm	*f*	$E_{\max}^{\mathrm{static}}$	ξ
Water			
50	0.94	−77.6	0.13
100	0.94	−68.3	0.11
200	0.92	−62.6	0.10
Acetonitrile			
50	0.95	−41.1	0.07
100	0.94	−27.4	0.05
200	0.90	−13.2	0.02

## Supplementary Materials

The following supporting information are available now: https://www.mdpi.com/article/10.3390/molecules27196402/s1, Figure S1: Results from EPR measurements of dissolved GT in the concentration range of 1 mM to 50 mM in water; Figure S2: Calibration line from EPR measurements of dissolved GT in the concentration range of 1 mM to 50 mM in water; Figure S3: EPR measurements of CPG-PEI800-GT in water; Figure S4: EPR measurements of CPG-PEI25000-GT in water; Figure S5: $T_{1}$ values of water and acetonitrile in contact with dissolved TEMPO; Figure S6: Calculated ODNP leakage factors of water water and acetonitrile in contact with dissolved TEMPO; Figure S7: Calculated ODNP leakage factors of water and acetonitrile in contact with synthesized CPG radical matrices; Figure S8: Continuous-flow ODNP measurements with CPG-PEI25000-GT (pore size 50 nm) and acetonitrile.

## Appendix A. Synthesis of CPG Radical Matrices

The chemicals used in the following synthesis are listed in Table A1. Two syntheses are presented, which provide different couplings to the support material: (1) without additional linker; (2) with PEI- and BDGE-linker.

(1) Aminopropyl-CPGs (350 mg) were added to ethanol (5 × 2.0 mL) and centrifuged in each case (5 min at 2500 rpm). The supernatant was removed. GT (399.56 mg, 0.5 mM) was dissolved in methanol. This radical solution was added to previously prepared CPGs and the reaction mixture was rotated in a tube rotator for 24 h at room temperature. Water (5 × 15 mL) was added and CPGs were separated by filtration. The product was stored in water (2.0 mL) at 277 K.

(2) Aminopropyl-CPGs (350 mg) were added to ethanol (5 × 2.0 mL) and centrifuged in each case (5 min at 2500 rpm). The supernatant was removed. BDGE (283.15 mg) was dissolved in methanol (3.5 mL) and dry aminopropyl-CPGs were added. The reaction mixture was rotated in a tube rotator for 24 h at room temperature. Water (5 × 15 mL) as well as ethanol (2 × 15 mL) was added and centrifuged in each case (5 min at 2500 rpm). Polyethyleneimine (molecular mass of 800 g mol${}^{-1}$, 280 mg, 100 mM; molecular mass of 25,000 g mol${}^{-1}$, 8.75 g, 20 mM) was dissolved in methanol and the previously BDGE-grafted CPGs were added. The reaction mixture was rotated in a tube rotator for 24 h at room temperature. Water (5 × 15 mL) as well as afterwards ethanol (2 × 15 mL) was added and centrifuged in each case (5 min at 2500 rpm). Finally, GT (399.56 mg, 0.5 mM) was dissolved in methanol. This radical solution was added to prepared PEI-grafted CPGs and the reaction mixture was rotated in a tube rotator for 24 h at room temperature. Water (5 × 15 mL) was added and CPGs were separated by filtration. The product was stored in water (2.0 mL) at 277 K.

**Table A1:** Chemicals used in the presented immobilization procedure of GT onto aminopropyl-functionalized CPGs are listed with the respective suppliers and purities.

Chemical	Supplier	Purity
aminopropyl-CPGs	Biosearch Technologies	
GT	TCI	>0.95 g g${}^{-1}$
polyethyleneimine 25,000 g mol${}^{-1}$	Sigma Aldrich	>0.99 g g${}^{-1}$
polyethyleneimine 800 g mol${}^{-1}$		>0.98 g g${}^{-1}$
1,4-butanediol diglycidyl ether		>0.95 g g${}^{-1}$
methanol		>0.99 g g${}^{-1}$
ethanol		>0.99 g g${}^{-1}$
acetonitrile		>0.99 g g${}^{-1}$

## Appendix B. Experimental Procedures

### Appendix B.1. EPR Measurements

A MicroESR-X-Band spectrometer from Bruker was used to determine the radical loading of synthesized CPG radical matrices. EPR measurements were performed using microcapillaries from Blaubrand intramark and were sealed with Leica Microsystems Critoseal capillary tube sealant. Sample volume was around 4 μL. The radical matrix was previously stored in a large supernatant (20 mL) of acetonitrile or water for 24 h. The entire sample volume was filled with the radical matrix in static contact with the liquid to be investigated and the supernatant was removed carefully. The following parameters were used: microwave power 15 mW, modulation coil amplitude 1.0 G, receiver gain 12 dB and an average of 16 scans. The relative error in the results of our EPR measurements is less than 2%. The accuracy in quantification of GT with the obtained calibration line of dissolved GT in water, which is presented in the Supporting Information, is around 97%. We estimate that the relative error of presented immobilized GT concentration values is 3%.

### Appendix B.2. Inversion Recovery NMR Measurements

${}^{1}$H NMR measurements were performed using a 1 T benchtop NMR spectrometer (Spinsolve carbon, proton frequency of 43 MHz) manufactured by Magritek. 2.5 mm outer diameter special microprobe NMR tubes from Norell were used to minimize the sample volume. After the liquid and radical matrix to be examined was transferred into the NMR tubes, the supernatant was removed after brief centrifugation. Parameters of the inversion recovery measurements were set with the standard protocol supplied by Magritek, taking care that the delay between the individual measurements was chosen sufficiently high for complete equilibration of the nuclear spins, as well as the maximum inversion time. An averaging of 8 scans was used. The relative error in successive $T_{1}$ measurements is less than 1% in this work, but the relative errors in the quantification of NMR data is usually around 2%. Therefore, we estimate that the relative error of the presented $T_{1}$ values is 2%.

### Appendix B.3. Static ODNP Measurements

Hyperpolarization is always generated with an ENDOR probehead (EN4148X-MD4) from Bruker at 0.35 T in the Halbach magnet. The same microcapillaries as described for the EPR measurements were used, and the sample preparation was carried out similarly. Samples for ODNP measurements with varying pH were prepared using a 0.2 M hydrochloric acid solution and ultrapure water (produced in our laboratory with an Elix Essential 5 purification system from Merck Millipore). Dilution and also the pH measurement were performed with an 848 Titrino plus system from Metrohm. All measurements presented here were performed five times, with preparation of a new sample each time. The following parameters were used: microwave frequency was 9.67 GHz, microwave power adjusted in the range between 0.5 and 2.5 W, and single-scan acquisition. An average of 16 scans was used for the thermally polarized NMR measurements. Samples were irradiated for period of about five times the $T_{1}^{\mathrm{RM}}$ of the liquid in static contact with the CPG radical matrices. In ODNP measurements with varying pH, the microwave power was adjusted to 6 W. Static enhancements were calculated using the integral of the hyperpolarized signal (microwave on) divided by the integral of the thermally polarized signal (microwave off) that were measured directly in the ENDOR probehead.

### Appendix B.4. Flow ODNP Measurements

NMR data were always acquired with the 1 T benchtop NMR spectrometer in flow mode. NMR measurements are acquired with short acquisition times of around 400 ms. The delay between individual NMR scans was set to 2 s, which was used to ensure a full exchange of the liquid in the active volume of the RF coil even at low flow rates. All measurements presented here were performed three times. The following parameters were used: microwave frequency was 9.67 GHz, microwave power was adjusted in a range between 0.5 and 6.1 W and single-scan acquisition. An average of 16 scans was used for the thermally polarized NMR measurements. An HPLC pump manufactured by Flusys was used to set up the liquid flow, which is equipped with a mass flow meter (Mini CORI-Flow) purchased from Bronkhorst. Hyperpolarization was generated in the ENDOR probehead in the 0.35 T Halbach magnet in the first part of the setup. Flow enhancements were calculated using the integral of the hyperpolarized signal (microwave on) divided by the integral of the thermally polarized signal (microwave off) that were measured in the benchtop NMR spectrometer.

## References

[1] Natterer J., Bargon J. (1997). Parahydrogen induced polarization. Prog. Nuclear Magn. Resonan. Spectrosc..

[2] Bowers C.R., Weitekamp D.P. (1987). Parahydrogen and synthesis allow dramatically enhanced nuclear alignment. J. Am. Chem. Soc..

[3] Münnemann K., Spiess H.W. (2011). The art of signal enhancement. Nat. Phys..

[4] Appelt S., Baranga A.B.A., Erickson C.J., Romalis M.V., Young A.R., Happer W. (1998). Theory of spin-exchange optical pumping of 3He and 129Xe. Phys. Rev. A.

[5] Walker T.G., Happer W. (1997). Spin-exchange optical pumping of noble-gas nuclei. Rev. Mod. Phys..

[6] Carver T.R., Slichter C.P. (1956). Experimental Verification of the Overhauser Nuclear Polarization Effect. Phys. Rev..

[7] Ardenkjaer-Larsen J.H., Fridlund B., Gram A., Hansson G., Hansson L., Lerche M.H., Servin R., Thaning M., Golman K. (2003). Increase in signal-to-noise ratio of >10,000 times in liquid-state NMR. Proc. Natl. Acad. Sci. USA.

[8] Maly T., Debelouchina G.T., Bajaj V.S., Hu K.N., Joo C.G., Mak-Jurkauskas M.L., Sirigiri J.R., van der Wel P.C.A., Herzfeld J., Temkin R.J. (2008). Dynamic nuclear polarization at high magnetic fields. J. Chem. Phys..

[9] Gitti R., Wild C., Tsiao C., Zimmer K., Glass T.E., Dorn H.C. (1988). Solid/liquid intermolecular transfer of dynamic nuclear polarization. Enhanced flowing fluid proton NMR signals via immobilized spin labels. J. Am. Chem. Soc..

[10] Dorn H., Gitti R., Tsai K., Glass T. (1989). The flow transfer of a bolus with 1H dynamic nuclear polarization from low to high magnetic fields. Chem. Phys. Lett..

[11] Tsai K.H., Dorn H.C. (1990). A model for establishing the ultimate enhancements (A infinity) in the low to high magnetic field transfer dynamic nuclear polarization experiment. Appl. Magn. Reson..

[12] Dorn H.C., Glass T.E., Gitti R., Tsai K.H. (1991). Transfer of 1H and 13C dynamic nuclear polarization from immobilized nitroxide radicals to flowing liquids. Appl. Magn. Reson..

[13] Lingwood M.D., Siaw T.A., Sailasuta N., Ross B.D., Bhattacharya P., Han S. (2010). Continuous flow Overhauser dynamic nuclear polarization of water in the fringe field of a clinical magnetic resonance imaging system for authentic image contrast. J. Magn. Reson..

[14] Lingwood M.D., Sederman A.J., Mantle M.D., Gladden L.F., Han S. (2012). Overhauser dynamic nuclear polarization amplification of NMR flow imaging. J. Magn. Reson..

[15] McCarney E.R., Armstrong B.D., Lingwood M.D., Han S. (2007). Hyperpolarized water as an authentic magnetic resonance imaging contrast agent. Proc. Natl. Acad. Sci. USA.

[16] McCarney E.R., Han S. (2008). Spin-labeled gel for the production of radical-free dynamic nuclear polarization enhanced molecules for NMR spectroscopy and imaging. J. Magn. Reson..

[17] Ebert S., Amar A., Bauer C., Kölzer M., Blümler P., Spiess H.W., Hinderberger D., Münnemann K. (2012). A Mobile DNP Polarizer for Continuous Flow Applications. Appl. Magn. Reson..

[18] Wang X., Isley W.C., Salido S.I., Sun Z., Song L., Tsai K.H., Cramer C.J., Dorn H.C. (2015). Optimization and prediction of the electron-nuclear dipolar and scalar interaction in 1H and 13C liquid state dynamic nuclear polarization. Chem. Sci..

[19] Denysenkov V., Terekhov M., Maeder R., Fischer S., Zangos S., Vogl T., Prisner T.F. (2017). Continuous-flow DNP polarizer for MRI applications at 1.5 T. Sci. Rep..

[20] Kircher R., Hasse H., Münnemann K. (2021). High Flow-Rate Benchtop NMR Spectroscopy Enabled by Continuous Overhauser DNP. Anal. Chem..

[21] Keller T.J., Laut A.J., Sirigiri J., Maly T. (2020). High-resolution Overhauser dynamic nuclear polarization enhanced proton NMR spectroscopy at low magnetic fields. J. Magn. Reson..

[22] Yoder J.L., Magnelind P.E., Espy M.A., Janicke M.T. (2018). Exploring the Limits of Overhauser Dynamic Nuclear Polarization (O-DNP) for Portable Magnetic Resonance Detection of Low Gamma Nuclei. Appl. Magn. Reson..

[23] Keller T.J., Maly T. (2021). Overhauser Dynamic Nuclear Polarization Enhanced Two-Dimensional Proton NMR Spectroscopy at Low Magnetic Fields. Magn. Reson..

[24] Kiss S.Z., MacKinnon N., Korvink J.G. (2021). Microfluidic Overhauser DNP chip for signal-enhanced compact NMR. Sci. Rep..

[25] Fyfe C.A., Cocivera M., Damji S.W.H. (1978). Flow and stopped-flow nuclear magnetic resonance investigations of intermediates in chemical reactions. Acc. Chem. Res..

[26] Haw J.F., Glass T.E., Hausler D.W., Motell E., Dorn H.C. (1980). Direct coupling of a liquid chromatograph to a continuous flow hydrogen nuclear magnetic resonance detector for analysis of petroleum and synthetic fuels. Anal. Chem..

[27] Godejohann M., Preiss A., Mügge C. (1998). Quantitative Measurements in Continuous-Flow HPLC/NMR. Anal. Chem..

[28] Maiwald M., Fischer H.H., Kim Y.K., Hasse H. (2003). Quantitative on-line high-resolution NMR spectroscopy in process engineering applications. Anal. Bioanal. Chem..

[29] Maiwald M., Steinhof O., Hasse H. (2007). Online-NMR-Spektroskopie—Mischungen messen im Fluss. Nachr. Chem..

[30] Wei R., Hall A.M.R., Behrens R., Pritchard M.S., King E.J., Lloyd-Jones G.C. (2021). Stopped-Flow 19F NMR Spectroscopic Analysis of a Protodeboronation Proceeding at the Sub-Second Time-Scale. Eur. J. Org. Chem..

[31] Friebel A., von Harbou E., Münnemann K., Hasse H. (2020). Online process monitoring of a batch distillation by medium field NMR spectroscopy. Chem. Eng. Sci..

[32] Friebel A., von Harbou E., Münnemann K., Hasse H. (2019). Reaction Monitoring by Benchtop NMR Spectroscopy Using a Novel Stationary Flow Reactor Setup. Ind. Eng. Chem. Res..

[33] Thomson C.G., Jones C.M.S., Rosair G., Ellis D., Marques-Hueso J., Lee A.L., Vilela F. (2020). Continuous-flow synthesis and application of polymer-supported BODIPY Photosensitisers for the generation of singlet oxygen; process optimised by in-line NMR spectroscopy. J. Flow Chem..

[34] Dorn H., Wang J., Allen L., Sweeney D., Glass T. (1988). Flow dynamic nuclear polarization, a novel method for enhancing NMR signals in flowing fluids. J. Magn. Reson. (1969).

[35] Lingwood M.D., Han S. (2011). Solution-State Dynamic Nuclear Polarization. Annual Reports on NMR Spectroscopy.

[36] Cheng T., Mishkovsky M., Junk M.J.N., Münnemann K., Comment A. (2016). Producing Radical-Free Hyperpolarized Perfusion Agents for In Vivo Magnetic Resonance Using Spin-Labeled Thermoresponsive Hydrogel. Macromol. Rapid Commun..

[37] Dollmann B.C., Junk M.J.N., Drechsler M., Spiess H.W., Hinderberger D., Münnemann K. (2010). Thermoresponsive, spin-labeled hydrogels as separable DNP polarizing agents. Phys. Chem. Chem. Phys..

[38] Bahulekar R., Ayyangar N., Ponrathnam S. (1991). Polyethyleneimine in immobilization of biocatalysts. Enzyme Microb. Technol..

[39] Virgen-Ortíz J.J., dos Santos J.C.S., Berenguer-Murcia Á., Barbosa O., Rodrigues R.C., Fernandez-Lafuente R. (2017). Polyethylenimine: A very useful ionic polymer in the design of immobilized enzyme biocatalysts. J. Mater. Chem. B.

[40] de Melo R.R., Alnoch R., Sousa A., Sato H.H., Ruller R., Mateo C. (2019). Cross-Linking with Polyethylenimine Confers Better Functional Characteristics to an Immobilized beta-glucosidase from Exiguobacterium antarcticum B7. Catalysts.

[41] Ghriga M.A., Grassl B., Gareche M., Khodja M., Lebouachera S.E.I., Andreu N., Drouiche N. (2019). Review of recent advances in polyethylenimine crosslinked polymer gels used for conformance control applications. Polym. Bull..

[42] Overhauser A.W. (1953). Polarization of Nuclei in Metals. Phys. Rev..

[43] Slichter C.P. (2014). The discovery and renaissance of dynamic nuclear polarization. Rep. Prog. Phys..

[44] Slichter C.P. (2010). The discovery and demonstration of dynamic nuclear polarization—A personal and historical account. Phys. Chem. Chem. Phys..

[45] Hausser K., Stehlik D., Waugh J.S. (1968). Dynamic Nuclear Polarization in Liquids. Advances in Magnetic Resonance.

[46] Ravera E., Luchinat C., Parigi G. (2016). Basic facts and perspectives of Overhauser DNP NMR. J. Magn. Reson..

[47] Abragam A., Goldman M. (1978). Principles of dynamic nuclear polarisation. Rep. Prog. Phys..

[48] Gafurov M., Denysenkov V., Prandolini M.J., Prisner T.F. (2012). Temperature Dependence of the Proton Overhauser DNP Enhancements on Aqueous Solutions of Fremy’s Salt Measured in a Magnetic Field of 9.2 T. Appl. Magn. Reson..

[49] Sezer D., Prandolini M.J., Prisner T.F. (2009). Dynamic nuclear polarization coupling factors calculated from molecular dynamics simulations of a nitroxide radical in water. Phys. Chem. Chem. Phys..

[50] Sezer D. (2014). Rationalizing Overhauser DNP of nitroxide radicals in water through MD simulations. Phys. Chem. Chem. Phys..

[51] Küçük S.E., Neugebauer P., Prisner T.F., Sezer D. (2015). Molecular simulations for dynamic nuclear polarization in liquids: A case study of TEMPOL in acetone and DMSO. Phys. Chem. Chem. Phys..

[52] Luchinat C., Parigi G. (2008). Nuclear Relaxometry Helps Designing Systems for Solution DNP on Proteins. Appl. Magn. Reson..

[53] Neugebauer P., Krummenacker J.G., Denysenkov V.P., Helmling C., Luchinat C., Parigi G., Prisner T.F. (2014). High-field liquid state NMR hyperpolarization: A combined DNP/NMRD approach. Phys. Chem. Chem. Phys..

[54] Höfer P., Parigi G., Luchinat C., Carl P., Guthausen G., Reese M., Carlomagno T., Griesinger C., Bennati M. (2008). Field Dependent Dynamic Nuclear Polarization with Radicals in Aqueous Solution. J. Am. Chem. Soc..

[55] Bennati M., Luchinat C., Parigi G., Türke M.T. (2010). Water 1H relaxation dispersion analysis on a nitroxide radical provides information on the maximal signal enhancement in Overhauser dynamic nuclear polarization experiments. Phys. Chem. Chem. Phys..

[56] Hyde J.S., Chien J.C.W., Freed J.H. (1968). Electron–Electron Double Resonance of Free Radicals in Solution. J. Chem. Phys..

[57] Türke M.T., Bennati M. (2011). Saturation factor of nitroxide radicals in liquid DNP by pulsed ELDOR experiments. Phys. Chem. Chem. Phys..

[58] Enkin N., Liu G., del Carmen Gimenez-Lopez M., Porfyrakis K., Tkach I., Bennati M. (2015). A high saturation factor in Overhauser DNP with nitroxide derivatives: The role of 14N nuclear spin relaxation. Phys. Chem. Chem. Phys..

[59] Wind R.A., Ardenkjær-Larsen J.H. (1999). 1H DNP at 1.4 T of Water Doped with a Triarylmethyl-Based Radical. J. Magn. Reson..

[60] Neugebauer P., Krummenacker J.G., Denysenkov V.P., Parigi G., Luchinat C., Prisner T.F. (2013). Liquid state DNP of water at 9.2 T: An experimental access to saturation. Phys. Chem. Chem. Phys..

[61] Neudert O., Zverev D.G., Bauer C., Blümler P., Spiess H.W., Hinderberger D., Münnemann K. (2012). Overhauser DNP and EPR in a Mobile Setup: Influence of Magnetic Field Inhomogeneity. Appl. Magn. Reson..

[62] Armstrong B.D., Han S. (2009). Overhauser Dynamic Nuclear Polarization To Study Local Water Dynamics. J. Am. Chem. Soc..

[63] Armstrong B.D., Han S. (2007). A new model for Overhauser enhanced nuclear magnetic resonance using nitroxide radicals. J. Chem. Phys..

[64] Gallops C.E., Yu C., Ziebarth J.D., Wang Y. (2019). Effect of the Protonation Level and Ionic Strength on the Structure of Linear Polyethyleneimine. ACS Omega.

