# Supplementary material for: Functionalized Controlled Porous Glasses for Producing Radical-Free Hyperpolarized Liquids by Overhauser DNP
Source: Molecules. 2022 Sep 28;27(19):6402. doi: 10.3390/molecules27196402 (PMC9572983; doi:10.3390/molecules27196402)
Supplement: Supplementary file 1 [file molecules-27-06402-s001.zip › molecules-1894306-supplementary.pdf]

molecules

Article

Supporting Information: Functionalized Controlled Porous Glasses for producing radical-free hyperpolarized liquids by Overhauser DNP

Raphael Kircher, Sarah Mross, Hans Hasse and Kerstin Münnemann*

Laboratory of Engineering Thermodynamics, University of Kaiserslautern, Kaiserslautern 67663, Germany
* kerstin.muennemann@mv.uni-kl.de

1. Quantification of Radicals by EPR Spectroscopy

EPR spectra were recorded with a MicroESR-X-Band spectrometer from Bruker. EPR spectra of dissolved 4-glycidyloxy-2,2,6,6-tetramethylpiperidine-1-oxyl (GT) in water are shown in Figure S1. Integrals of absorption spectra were used to generate a calibration line (y=a+b*x; R^2 = 0.97; see Figure S2) that was used to calculate the amount of immobilized GT of synthesized CPG radical matrices. EPR measurements were performed using microcapillaries from Blaubrand intramark with an inner diameter of 1 mm with a sample volume of 4 µL and were sealed with Leica Microsystems Critoseal capillary tube sealant. The following EPR acquisition parameters were used: microwave power 15 mW, modulation coil amplitude 1.0 G, receiver gain 12 dB and an average of 16 scans. This set of EPR parameters was also used in all following EPR measurements of synthesized CPGs in order to allow the quantitative evaluation of radical concentrations. The parameters are adjusted to higher concentrations of GT and thus, digitalization noise is visible for small concentrations of immobilized GT of radical matrices of the type CPG-PEI800-GT.

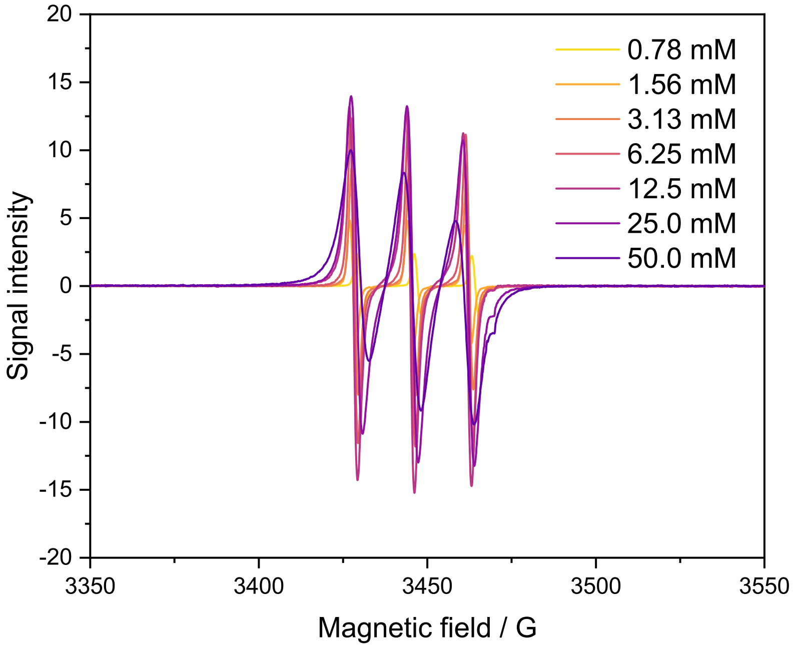

Figure S1. Results from EPR measurements of dissolved GT in the concentration range of 1 mM to 50 mM in water.

check for updates

Citation: Kircher, R.; Mross, S.; Hasse, H.; Münnemann, K. Supporting Information: Functionalized Controlled Porous Glasses for producing radical-free hyperpolarized liquids by Overhauser DNP. *Molecules* **2022**, *27*, 6402. https://doi.org/10.3390/molecules27196402

Received: 16 August 2022
Accepted: 25 September 2022
Published: 28 September 2022

Publisher's Note: MDPI stays neutral with regard to jurisdictional claims in published maps and institutional affiliations.

Copyright: © 2022 by the authors. Licensee MDPI, Basel, Switzerland. This article is an open access article distributed under the terms and conditions of the Creative Commons Attribution (CC BY) license (https://creativecommons.org/licenses/by/4.0/).

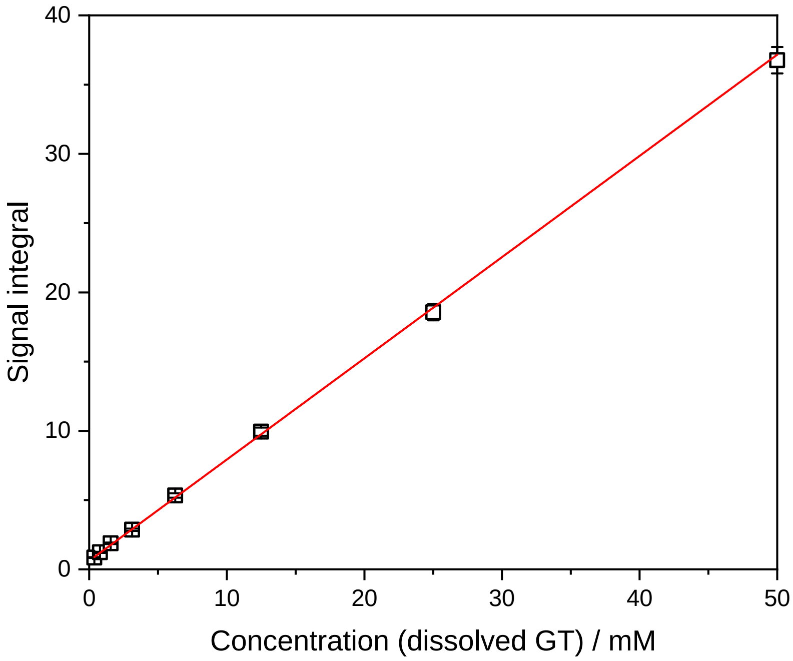

Figure S2. Calibration line from EPR measurements of dissolved GT (shown in Figure S1) in the concentration range of 1 mM to 50 mM in water.

To study the amount of immobilized GT of synthesized radical matrices, 100 mg of each radical matrix was previously stored in a large supernatant (20 mL) of acetonitrile or water for 24 h. The entire sample volume of 4 µL was filled with the radical matrix in static contact with the liquid to be investigated and the supernatant was removed carefully after brief centrifugation.

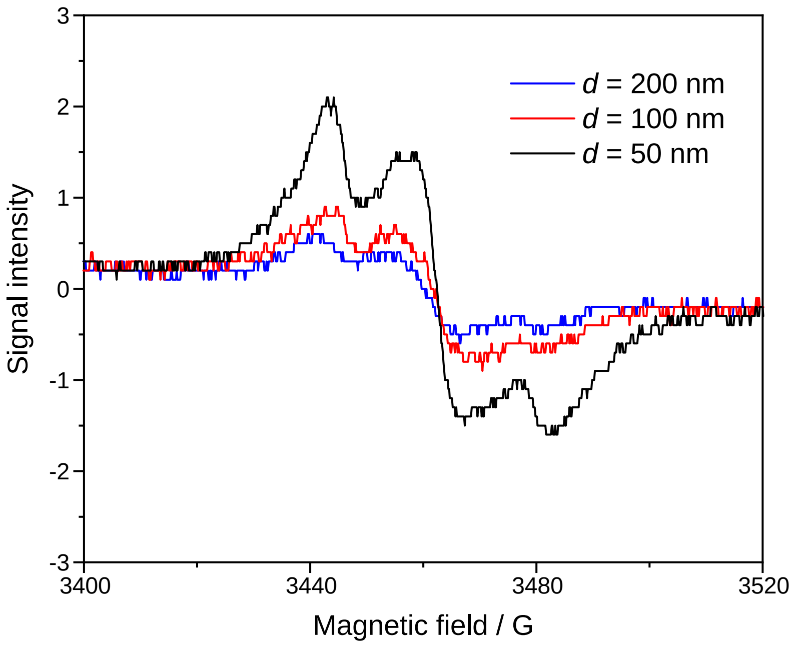

Figure S3. Results from EPR measurements of the synthesized CPGs of the type CPG-PEI800-GT with different pore size *d* of CPGs in water.

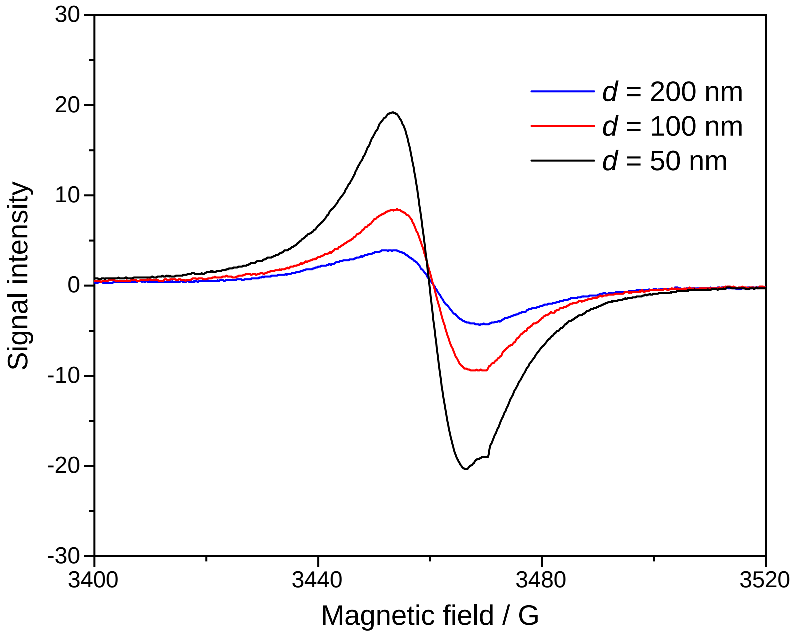

Figure S4. Results from EPR measurements of the synthesized CPGs of the type CPG-PEI25000-GT with different pore size *d* of CPGs in water.

Figure S3 and Figure S4 show the first derivative of the EPR spectra of synthesized CPG radical matrices with different pore sizes of 50, 100, and 200 nm with the coupled polyethyleneimine (PEI) polymer PEI800 and PEI25000 in water. The amount of immobilized GT was calculated by comparison of EPR integrals with aqueous solutions of 4-glycidyloxy-TEMPO (GT) of known concentrations, see Figure S2 and calculated concentration values are listed in the main manuscript in Table 1. The immobilization of GT leads to a significant broadening of the measured EPR lines. This effect combined with Heisenberg spin exchange at high radical loading accomplished with the high molecular weight polymer results in a single broad line for radical matrices of the type CPG-PEI25000-GT in the absorption spectrum.

2. Leakage Factors with Dissolved TEMPO-radicals

Leakage factors were calculated using Equation 2 in the main manuscript. This ODNP parameter was directly accessible via NMR inversion recovery measurements that were performed with a 1 T benchtop NMR spectrometer from Magritek. 2.5 mm outer diameter special microprobe NMR tubes from Norell were used to minimize the sample volume. Parameters of the inversion recovery measurements were set with the standard protocol supplied by Magritek, taking care that the delay between the individual measurements was chosen sufficiently high for complete equilibration of the nuclear spins, as well as the maximum inversion time. An average of 8 scans was used.

T_1 values of acetonitrile and water in static contact with dissolved TEMPO radicals are shown in Figure S5. In this work, a specially functionalized TEMPO radical was used, namely 4-glycidyloxy-2,2,6,6-tetramethylpiperidine-1-oxyl (GT). We have checked the relaxation behavior of acetonitrile and water which were doped with dissolved 4-hydroxy-TEMPO (HT), 4-amino-TEMPO (AT), and GT of different concentrations.

The relaxation efficiency for water and acetonitrile does not change with the different functionalization of TEMPO, as expected, because the functionalization is far from the unpaired electron delocalized via the N-O bond, which is additionally shielded by the neighboring methyl groups. Figure S6 shows calculated leakage factors in dependence of the radical concentration of dissolved TEMPO radicals in acetonitrile and water. For the relaxation behavior of acetonitrile and water with dissolved TEMPO-radicals, the leakage

factor is always better for water than for acetonitrile at a comparable concentration of TEMPO, which points to a preferred radical-target molecule interaction with water.

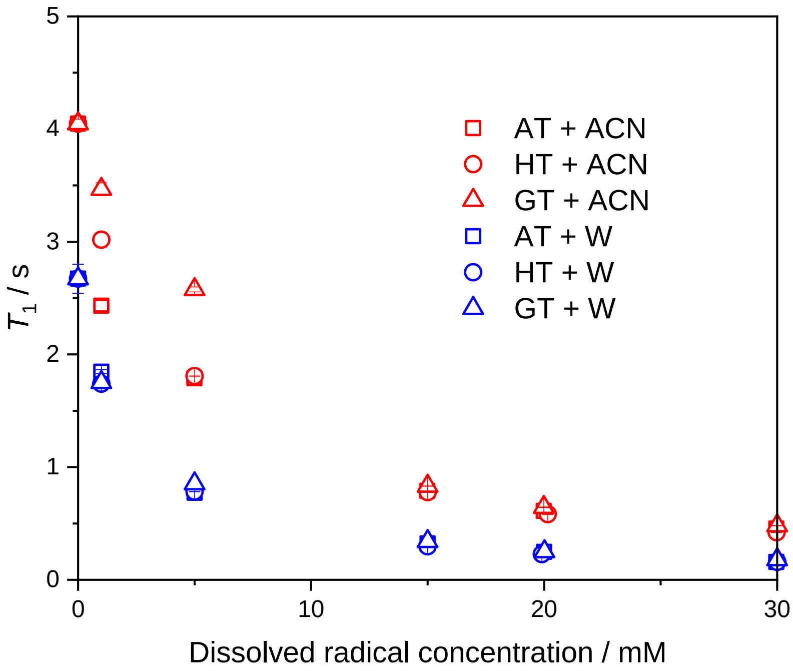

Figure S5. Results of inversion recovery measurements with water (W) and acetonitrile (ACN) in contact with dissolved TEMPO radicals, i.e. 4-amino-TEMPO (AT), 4-hydroxy-TEMPO (HT) and 4-glycidyloxy-TEMPO (GT). The symbols are experimental results and were obtained as the arithmetic mean of three individual samples. The error bars indicate the standard deviation.

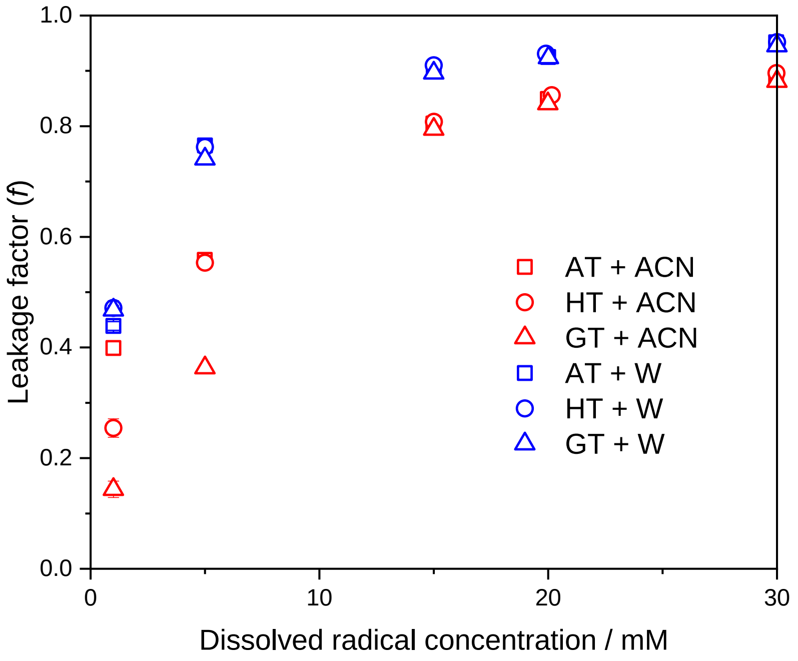

Figure S6. Results of calculated ODNP leakage factors with water (W) and acetonitrile (ACN) in contact with dissolved TEMPO radicals, i.e. 4-amino-TEMPO (AT), 4-hydroxy-TEMPO (HT) and 4-glycidyloxy-TEMPO (GT). The symbols are experimental results and were obtained as the arithmetic mean of three individual samples. The error bars indicate the standard deviation. Leakage factors are calculated with Equation 2 in the main manuscript.

3. Leakage Factors with CPG Radical Matrices

Sample preparation and NMR acquisition was carried out similarly to the experiments with dissolved radicals. After the liquid and radical matrix to be examined was transferred into the NMR tubes, the supernatant was removed after brief centrifugation. Figure S7 shows calculated leakage factors in dependence of the radical concentration of CPG radical matrices in acetonitrile and water, the corresponding T_1 values are listed in Table 2 in the main manuscript. The behavior of the leakage factor is influenced purely by the immobilized radical loading in the CPG radical matrices and no further effect of the pore size can be seen in the range of CPG pore diameters studied here.

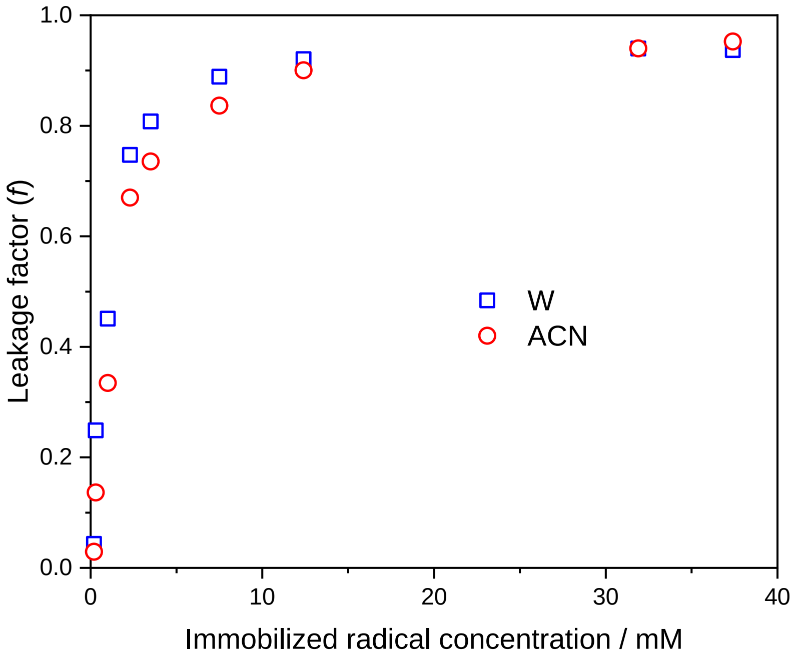

Figure S7. Results of calculated ODNP leakage factors versus radical concentration of synthesized CPG radical matrices in pure water (W) and acetonitrile (ACN). Leakage factors are calculated with T_1 values listed in Table 2 in the main manuscript and Equation 2 in the main manuscript.

4. Flow ODNP of Acetonitrile

Figure S8 shows results of continuous ODNP measurements with CPG-PEI25000-GT (pore size 50 nm) with acetonitrile, in which both the microwave power and the flow rate were varied. The highest flow rate corresponds to a superficial flow velocity of about 0.7 m s^{-1}. From ODNP data dependent on microwave power, E_{max}^{flow} values for the enhancement at infinite microwave power can be extracted by exponential extrapolation [1,2]. Figure S8 demonstrates that a large sensitivity improvement in continuous-flow benchtop NMR spectroscopy can be accomplished by ODNP also for acetonitrile. This is especially important in the fast flow-regime where the thermal NMR signal almost completely vanishes due to very short prepolarization times.

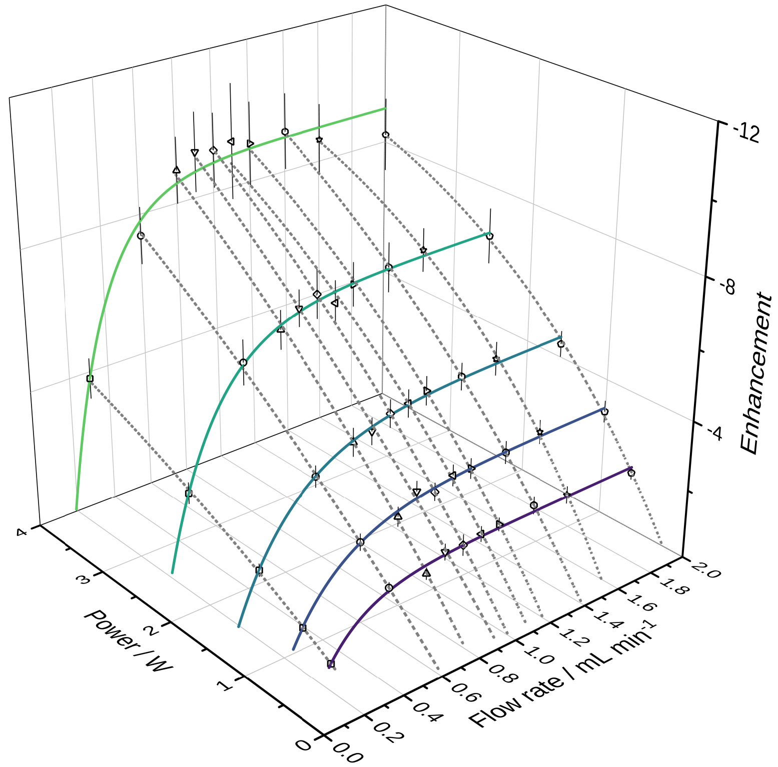

Figure S8. Results from continuous-flow ODNP measurements with acetonitrile obtained with CPG-PEI25000-GT (pore size 50 nm) versus applied microwave power and flow rate. The symbols are experimental results and were obtained as the arithmetic mean of the results of three individual samples. Solid lines: guide to the eye. Dashed lines: exponential fit [1,2].

Author Contributions: Conceptualization, R.K., H.H. and K.M.; methodology, R.K.; software, S.M. and R.K.; validation, S.M. and R.K.; investigation, R.K.; writing—original draft preparation, R.K.; writing—review and editing, R.K.,H.H. and K.M.; visualization, R.K. and S.M.; supervision, H.H. and K.M.; project administration, H.H. and K.M.; funding acquisition, K.M. and H.H. All authors have read and agreed to the published version of the manuscript.

Funding: The authors thank the German Research Foundation (DFG) for financial support within the Collaborative Research Center SFB 1527 "High Performance Compact Magnetic Resonance – HyPERiON".

Conflicts of Interest: The authors declare no conflict of interest.

References

1. Armstrong, B.D.; Han, S. Overhauser Dynamic Nuclear Polarization To Study Local Water Dynamics. *Journal of the American Chemical Society* **2009**, *131*, 4641–4647. https://doi.org/https://doi.org/10.1021/ja809259q.
2. Hausser, K.; Stehlik, D. Dynamic Nuclear Polarization in Liquids. In *Advances in Magnetic Resonance*; WAUGH, J.S., Ed.; Academic Press, 1968; Vol. 3, *Advances in Magnetic and Optical Resonance*, pp. 79–139. https://doi.org/https://doi.org/10.1016/B978-1-4832-3116-7.50010-2.
